# Computational protocol for modeling supported nanoparticle catalysts with strong metal-support interactions

**DOI:** 10.1016/j.xpro.2026.104655

**Published:** 2026-06-29

**Authors:** Gerardo Valadez Huerta, Michihisa Koyama

**Affiliations:** 1 Institute for Aqua Regeneration, Shinshu University, Nagano, Nagano 380-8553, Japan

**Keywords:** Chemistry, Material sciences, Computer sciences

## Abstract

Modeling supported nanoparticle catalysts with strong metal-support interactions (SMSI) is challenging due to their high structural complexity. Here, we present a computational protocol that uses a modular and adaptive Python-based framework to achieve this goal. We describe steps for constructing supported nanoparticle models and exploring large configurational spaces of adsorption states. We then detail procedures for guiding the implementation of analysis workflows to identify catalytically relevant configurations by matching simulated and experimental infrared (IR) spectra.

For complete details on the use and execution of this protocol, please refer to G. Valadez Huerta et al.^1^

## Highlights

• Assemble supported nanoparticle catalyst models with strong metal-support interactions
• Build and optimize adsorption configurations and calculate dissociation pathways
• Match simulated and experimental vibrational spectra to identify key adsorption sites

## Abstract (editor highlights)

Publisher’s note: Undertaking any experimental protocol requires adherence to local institutional guidelines for laboratory safety and ethics.

## Graphical abstract

**Figure:**
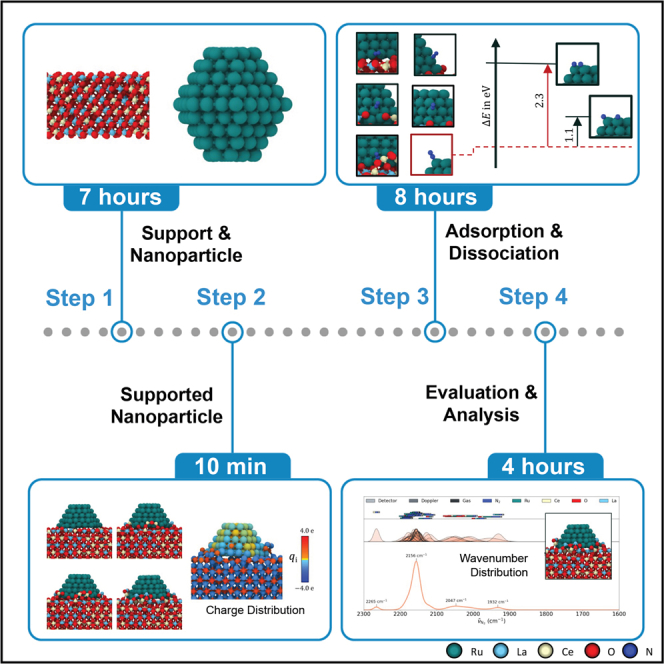

## Before you begin

The protocol below describes the modeling and simulation workflow developed for Ru/La_0_._5_Ce_0_._5_O_1_._75-x_ supported nanoparticles exhibiting strong metal–support interactions (SMSI), as used in the associated research article.^1^ By following this protocol, users can generate nanoparticle–support configurations with different degrees of reduction and SMSI-induced nanoparticle decoration, and systematically screen N_2_ adsorption states. The modular design of the code makes it straightforward to extend the workflow to other material combinations reported in the literature.^2^^,^^3^^,^^4^ The force field employed in this workflow is explicitly specified, allowing users to replace the PreFerred Potential (PFP)^5^ with other neural network potentials, classical force fields, or *ab initio* methods. The workflow is executed in serial, while parallelization depends on the implementation of the chosen force field. Finally, the protocol includes the implementation of the genetic algorithm (GA) used in the associated research article^1^ to match simulated and experimental IR spectra and to identify highly active sites. This implementation is provided as guidance for conducting similar analyses for other systems. Familiarity with Python and ASE^6^ is advantageous when adapting modules to introduce new material combinations.

### Innovation

The recent literature offers a variety of frameworks enabling the optimization of various catalyst classes, including alloy nanoparticles in vacuum,^7^ metal-metal oxide catalytic interfaces,^8^ supported clusters,^9^ and adsorbate coverage on supported clusters.^10^ However, none of these frameworks have been applied to supported nanoparticle catalysts with SMSI at the level of structural complexity addressed here. In contrast to previous approaches, the present workflow integrates experimental IR spectroscopy as a structural validation and interpretation tool for identifying catalytically relevant adsorption environments. In addition, existing approaches are generally based directly on density functional theory (DFT) and, thus, cannot feasibly sample the large configurational spaces, extensive surface reconstructions, and thousands of adsorption states required to capture SMSI. This protocol was designed to apply accurate neural network potentials, allowing users to construct large supported nanoparticles across different reduction degrees, generate SMSI-induced decoration configurations, and systematically evaluate adsorption. A further innovation is the adaptability of the framework. Although originally developed for Ru/La_0_._5_Ce_0_._5_O_1_._75-x_, the workflow can be extended to other supports, nanoparticle configurations, adsorbates, or alternative models for interatomic forces and energies. This flexibility makes the protocol applicable to a wide range of supported catalyst systems. By enabling the modeling of high-complexity SMSI systems, the protocol provides a framework for computational exploration of supported nanoparticle catalysts.

### Prepare tools

**Timing: 30 min**

1. Install Python and Jupyter Notebook (JN)
  ***Note:*** Python 3.9 or higher is required. The workflow can also be converted to standalone Python scripts for use on Linux systems.
  a. Install the following Python modules: os, numpy, pathlib, random, ase, pandas, flare (mir-flare), statsmodels, shelve, pickle and vala_shinshu.
    ***Note:*** The *vala_shinshu* module is under active development and maintained in a private GitHub repository. For the purpose of this protocol, a stable version of the module compatible with the present workflow is included in the associated Mendeley Data repository (see Key Resources Table). Users should download the “ValaShinshu” directory and run the file “Install.ipynb” to install the module and its required dependencies.
2. Download the “SupplementaryDataP” directory from the Mendeley Data repository (see Supplementary Data P in the Key Resources Table).

***Note:*** Each step is executed within a directory tree containing multiple directory levels, with the code located in a subdirectory called *Python*. Because the code automatically searches across atomistic models to perform calculations or generate subsequent models as follows:

> for xyz in root.glob(globName)

the *globName* variable must be adjusted according to the directory depth. Directories to be read should be specified relative to the *Python* directory where the code is located. For example, if the directory to be read is *Data* and it is located at the same level as the *Python* directory, the *root* variable should be defined accordingly:

> root = Path(“../Data”)

If *Data* contains four subdirectories, the directory depth must be reflected consistently in the definition of *globName*

> globName = “∗/∗/∗/∗”

The ∗ can be replaced with a specific subdirectory name, in which case the code will run using only the files within that subdirectory.

***Note:*** The current code works with PFP^5^ version 1.1.0 within the Matlantis^11^ Environment. To use a different version or an alternative potential, consult the corresponding potential and ASE documentation, verify compatibility with ASE, replace all “PFP” strings with the appropriate identifiers, and adapt the indicated lines accordingly:

 > from pfp_api_client.pfp.calculators.ase_calculator import ASECalculator

 > from pfp_api_client.pfp.estimator import Estimator

 > estimator = Estimator(model_version=“v1.1.0”)

 > calculator = ASECalculator(estimator)

For instance, an implementation using the CHGNet potential can be defined as follows:

 > from chgnet.model.dynamics import CHGNetCalculator

 > calculator = CHGNetCalculator()

Within the protocol, the bulk calculation step (see Section “Support Modeling & Simulation: Bulk”) includes an example implementation that allows the use of either PFP or CHGNet,^12^ enabling users to select the desired potential. The remaining parts of the workflow can be adapted in a similar manner. This approach is also readily applicable to other all-purpose interatomic potentials, such as MACE,^13^ UMA^14^ or LASP.^15^

***Note:*** Atomistic models are stored as XYZ files. Structures generated directly with ASE and Python are written to the *Data* directory. Models obtained after structure optimization, molecular dynamics (MD), or vibrational calculations are saved in the *Results* directory, while evaluated properties are written to the *Eval* directory. Additional directory levels are used to specify, for example, the calculation method (e.g. */PFP*) or individual adsorption configurations (*/1*, */2*). Rendered images are stored in *Figures/SourceDirectory*, where *SourceDirectory* refers to *Data*, *Results*, or *Eval*, depending on the origin of the source files. In all cases, the subdirectory structure of the source files is preserved.

***Note:*** Variable names throughout the codes are kept consistent in both name and purpose and are introduced only once in the protocol to avoid repetitiveness.

***Note:*** All diagrams generated using the Python matplotlib module are saved at a resolution of 300 dpi when exported as PNG and are also saved in PDF and SVG formats, although only the PNG file names are referenced in the protocol.

***Note:*** The reported timing for major steps refers to the computational time for a single catalyst configuration of the reference case study, evaluated on the Matlantis servers for steps requiring PFP. Non-PFP steps (in particular Ovito image generation) were executed on a local machine equipped with an Intel Core i7-10750H (10th Gen) CPU and 16 GB RAM. Time required for code adaptations is not included.

## Key resources table

**Table:** 

REAGENT or RESOURCE	SOURCE	IDENTIFIER
**Deposited data**		

Supplementary Data P	This Study	https://doi.org/10.17632/ssk54nxjnr.1
Supplementary Data A	Associated Research Article^1^	https://doi.org/10.17632/w7xjrr4fc3.1
Supplementary Data B	Associated Research Article^1^	https://doi.org/10.17632/w7xjrr4fc3.1
Code	Associated Research Article^1^	https://doi.org/10.17632/w7xjrr4fc3.1
vala_shinshu	Own created Python module for atomistic modeling	https://data.mendeley.com/preview/ssk54nxjnr?a=9edad0ad-6796-44a9-9916-cbb5351e887b
Materials Project Database	Jain et al.^16^	https://next-gen.materialsproject.org/

**Software and algorithms**		

PreFerred Potential version 1.1.0	Matlantis^5^^,^^11^	https://doi.org/10.1038/s41467-022-30687-9 https://matlantis.com/
Atomic Simulation Environment	Larsen et al.^6^	https://wiki.fysik.dtu.dk/ase/
FLARE	Vandermause et al.^17^	https://flare.readthedocs.io/
OVITO	OVITO GmbH^18^	https://www.ovito.org/
Python version 3.9	Python™	https://www.python.org/
Jupyter Notebook	Project Jupyter	https://jupyter.org/
NumPy	Harris et al.^19^	https://numpy.org/
Pandas	The pandas development team	https://pandas.pydata.org/
Statsmodels	Seabold and Perktold^20^	https://www.statsmodels.org/stable/index.html
Matplotlib	Hunter^21^	https://matplotlib.org/
CHGNet	Deng et al.^12^	https://github.com/CederGroupHub/chgnet
MS Word	Microsoft Corporation	https://www.microsoft.com/en-us/microsoft-365/word
MS PowerPoint	Microsoft Corporation	https://www.microsoft.com/en-us/microsoft-365/powerpoint

## Step-by-step method details

### Support modeling and simulation: Bulk

**Timing: 25 min**

**Figure 1:**
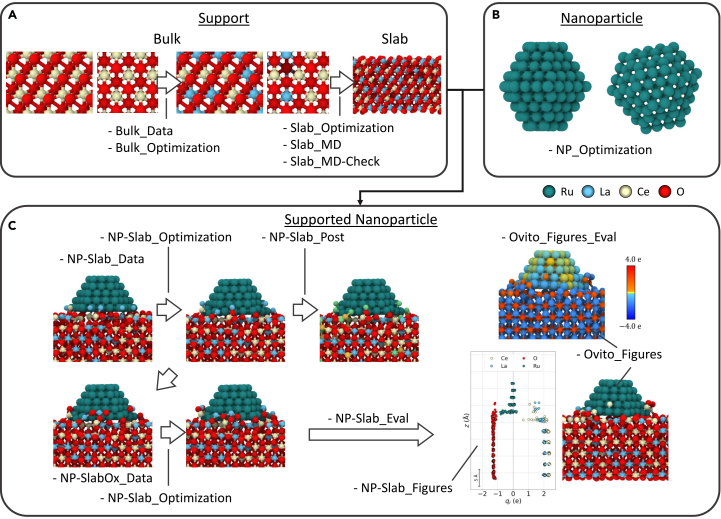
Workflow for the construction, optimization, and evaluation of supported nanoparticle models, illustrated by representative atomistic structures and resulting figures (A) Generation of the oxide support starting from bulk configurations, followed by slab modeling, structural optimization, and molecular dynamics simulations. (B) Construction and optimization of the metal nanoparticle in vacuum. (C) Assembly, optimization, and evaluation of the supported nanoparticle system.

Model and simulate the support bulk material. Relax all atomic positions and optimize the unit cell to obtain the equilibrium bulk configuration (see Figure 1A).

***Note:*** The working directory for this step is *SupplementaryDataP/Solids/Bulk/MetalOxides/Binary/*.

1. Prepare the base bulk models for the selected support and store them in the *Preparation* subdirectory

***Note:*** For the associated study on Ru/La_0_._5_Ce_0_._5_O_1_._75-x_, reference directories of the form *Preparation/La-Ce-O/La2Ce2O7/Cubic/111/4x4x3/XX* are already provided and base bulk models do not need to be created. The subdirectories XX = *Ce-1*, *La-1*, *SP-1*, and *SS-1* correspond to base models with ceria-rich (Ce), lanthanum-rich (La), superlattice (SP), and solid-solution (SS) cationic surfaces, respectively, as described in the associated research article.^1^ For the Ce, La, and SS cases, the underlying bulk configuration is a 4×4×3 CeO_2_ superlattice oriented along the (111) direction. For the SP case, a configuration with intercalated La and Ce layers is used. Users can adapt the directory structure and model definitions according to the support material targeted in their study. The base bulk models should be saved as *Basis.xyz*.

2. Prepare the final bulk models, perform optimization, and visualize the results
  a. Run *Bulk_Data.ipynb*
    ***Note:*** The code generates bulk models saved as *Bulk.xyz*. The upper layer of each bulk model corresponds to the cationic surface layer of the slab and is defined by the directory name and specified via the variable *surfCase*. For example, if *surfCase = “Ce”*, only models within subdirectories named Ce-∗ are generated. The code introduces vacancies consistent with the La_0_._5_Ce_0_._5_O_1_._75_ composition and searches for low-energy Ce–La solid-solution configurations (see the associated research article^1^ for details). The workflow can be adapted to other A_x_BᵧO_z_ metal oxides by replacing
    > MtName = ['Ce','La']
    with
    > MtName = ['A','B']
    Alternatively, other systems can be handled by providing own created *Bulk.xyz* files.
  b. Run *Bulk_Optimization.ipynb*
    ***Note:*** The code generates optimized bulk configurations saved as *Opt_PFP.xyz*. In addition, CSV files containing information on all optimized models are written to *Cell_PFP.csv* (lattice constants and angles) and *Opt_PFP.csv* (total energy, composition, and related properties).
  c. Run *Ovito_Figures.ipynb*
    ***Note:*** The code generates figures of the bulk models from different viewpoints (see Figure 1A). The directory containing the models must be specified manually via the following variable
    > case = 'Data' # 'Preparation', 'Data', 'Results'

### Support modeling and simulation: Slab

**Timing: 7 h**

Model and simulate the slab support. Relax all atomic positions and conduct molecular dynamics to obtain slab configurations (see Figure 1A).

***Note:*** The working directory for this step is *SupplementaryDataP/Solids/Slab/MetalOxides/Binary/*.

3. Prepare and optimize the slab models
  a. Run *Slab_Optimization.ipynb*

***Note:*** Slab models are generated from optimized bulk configurations located in the directory specified by *dirBulk*. The generated slab structures are saved as *Slab.xyz*, and the optimized slab models are stored as *Opt_PFP.xyz*.

4. MD Slab Equilibration
  a. Run *Slab_MD.ipynb*
    ***Note:*** To modify the molecular dynamics parameters, adjust the following variables:
    Here, *dt* is the time step in femtoseconds, *tequil* is the total number of equilibration steps, and *T* is the temperature in Kelvin. The last frame of the MD trajectory is saved as *Slab_Equil.xyz*.
    **CRITICAL:** The FLARE^17^ module may not install reliably under Python 3.10 or higher. For the MD simulations in this protocol, use Python 3.9. Alternatively, adapt the workflow to a different MD engine (e.g. LAMMPS^22^) or use the ASE’s^6^ molecular dynamics functionality.
    > dt = 0.5 # in fs
    > tequil = 200000 # in steps
    > T = 923 # in K
  b. Run *Slab-MD_Check.ipynb*
    ***Note:*** The automatic equilibration detection method of Chodera^23^ is applied, and figures similar to Figure S29 in the Supplementary Information of the associated research article^1^ are generated.
  c. Run Ovito_Figures.ipynb
    ***Note:*** The variable
    > simMod = 'PFP' # 'MD' or 'PFP'
    is used to switch between results obtained from structure optimization (“PFP”) and molecular dynamics simulations (“MD”).

### Nanoparticle modeling and simulation

**Timing: 1 min**

Model and optimize nanoparticles by relaxing all atomic positions (see Figure 1B)

***Note:*** The working directory for this step is *SupplementaryDataP/Solids/Nanoparticles/Metals*/Monometal/

5. Run *NP-Optimization.ipynb*

***Note:*** Nanoparticle models are read from VASP-generated structures located in the *Data* directory. These structures originate from the study by Nanba et al.^24^ If data for monometallic nanoparticles are prepared following the same directory structure, changing the variable

> MtName = 'Ru'

to the desired element is sufficient. Structures for other nanoparticles can also be generated from scratch, for example using ASE^6^ modules for creating clusters or nanoparticles. The corresponding Results directories follow the structure *Ru/RuXX/Structure/PFP* (e.g., *Ru/Ru238/HCP/PFP*). Relaxed nanoparticle structures are saved as *NP.xyz*.

6. Run *Ovito_Figures.ipynb*

***Note:*** The variables *MtName* and *Structure* specify which models are visualized. Setting both variables to '∗' visualizes all available models. Example rendered images are shown in Figure 1B.

### Supported nanoparticle modeling and simulation

**Timing: 10 min**

Model and optimize supported nanoparticles by assembling the slab and nanoparticle models and relaxing all atomic positions (see Figure 1C).

***Note:*** The working directory for this step is *SupplementaryDataP/InterF/SS/NP-Slab/Mt-MtOx/Mono-Binary*

7. Run *NP-Slab-Data.ipynb*

***Note:*** The code is divided into two sections: one for preparing the nanoparticle and one for assembling the nanoparticle and slab into the supported nanoparticle model. For nanoparticle preparation, the following variables define the directory structure used for the nanoparticle calculations:

 > MtName = “Ru”

 > NP_N = 238

 > NPStruct = “HCP”

 > NP_N_thr = 150

 > NPFacet = “001”

 > PCVk = [1] # [1,2]

Here, *MtName* specifies the metal element, *NP_N* the number of atoms in the nanoparticle, and *NPStruct* the nanoparticle structure. *NP_N_thr* defines a size threshold used during nanoparticle creation. For example, with the value of 150, a five-layer nanoparticle is retained. Together, these variables define the directory structure from which the nanoparticle model is read:

>read(f'{prefNPDir}/Results/{MtName}/{MtName}{str(NP_N)}/{NPStruct}/PFP/NP.xyz')

Furthermore, *NPFacet* describes the crystallographic plane oriented along the z direction that contacts the slab surface and is used only to construct the subdirectory name for the supported nanoparticle model, as automatic plane detection is not implemented. The meaning of *PCVk* is explained later in this note, but it is introduced here because it defines the number of nanoparticle instances generated. The code for assembling the supported nanoparticle model is highly general and enables the construction of supported nanoparticle models both with and without SMSI. This generality can be controlled by modifying the following variables:

 > updoVk = ['UP'] # ['UP','DO']

 > angleVk = [165] # [75,105,135,165]

 > Sublayers = [1] # [1,2]

 > xOVk = [50,100] # [0,25,50,75,100]

 > yCat = [50] # [25,50,75,100]

 > dzPznt = 0.025 # in Percentage

 > dxCat = 1.5 # in Å

Here, *updoVk* controls whether the nanoparticle is placed on the upper side of the slab (‘UP’) or on the underside (‘DO’). This functionality was not used in the associated research article^1^ but allows the construction of models for asymmetric slabs using a single slab model as reference. Moreover, *angleVk* specifies rotation angles applied to the nanoparticle around the surface normal and is used to identify the most stable nanoparticle orientation. *Sublayers* defines the number of cationic support layers considered for reduction. The variable *xOVk* controls the fraction of oxygen atoms retained during reduction, expressed as a percentage (100 corresponds to full oxidation, whereas 0 corresponds to full reduction). The variable *yCat* controls the fraction of cations taken from the support and placed on the lowest nanoparticle layers to generate artificial SMSI. A value of 100 indicates that all nanoparticle atoms in the selected layers are on-top occupied by support cations. *PCVk* specifies the number of lowest nanoparticle layers considered for this procedure. Finally, *dzPznt* sets the initial vertical separation between the nanoparticle and the slab as a percentage of the slab height, while *dxCat* defines the distance between nanoparticle atoms and the cations placed on-top of them. While the definitions and purposes of these variables may appear abstract, readers are referred to the associated research article^1^ for a more detailed explanation. Finally, the resulting subdirectory structure is generated from a combination of the nanoparticle and slab directory structures together with the selected assembly variables, where the assembled model is saved as *NP-Slab.xyz*.

**CRITICAL:** Carefully verify that the modeling behaves as intended. For example, ensure that cations are selected exclusively from the layers specified by *Sublayers*. If cations from deeper layers are unintentionally included, reduce the value of *dzPznt*, as excessively large values may cause atoms from lower layers to be incorrectly classified. Correct modeling of the supported nanoparticle configurations is critical, as all subsequent steps depend on these configurations.

8. Run *NP-Slab_Optimization.ipynb*

***Note:*** Optimized structures are saved as *Opt_PFP.xyz*. Because structure optimization can be time-consuming, only models that have not yet been optimized are processed by default. Setting the variable

> restart = True

forces all structures to be recalculated and overwrites existing results.

9. Run *NP-SlabOx_Data.ipynb*

***Note:*** In this step, the code reads the optimized supported nanoparticle structures from the previous step and introduces oxidation of the SMSI region using only cations generated during the nanoparticle–support assembly. The extent of oxidation is scaled to remain consistent with the effective reduction state of the cationic support, as defined by the number of cationic layers. The following variables control this procedure:

> NLayersSlab = 6

> dxCatO = 2.0 # in Å

> dz = 1.4 # in Å

Here, *NLayersSlab* defines the number of cationic layers in the slab and is used to scale the number of oxygen atoms relocated during oxidation relative to the number of cations decorating the nanoparticle. The variable *dxCatO* defines the lateral distance, in Å, between relocated oxygen atoms and the corresponding SMSI cations, while *dz* defines the vertical offset used to place oxygen atoms relative to the nanoparticle.

10. Run *NP-Slab_Optimization.ipynb*

***Note:*** Rerunning the code optimizes the configurations generated in the previous step.

11. Run *NP-Slab_Post.ipynb*

***Note:*** This code reads the optimized supported nanoparticle configurations and performs a Wigner–Seitz analysis of defects (see the associated research article^1^ for details). The results are saved as *WS0_PFP.xyz* and *WS_PFP.xyz* in the *Post* directory.

12. Run *NP-Slab_Eval.ipynb*

***Note:*** This code reads the optimized supported nanoparticle structures and the files containing the Wigner–Seitz analysis of defects and calculates various properties. The results are saved as *Opt_PFP.csv* and *Opt_PFP.json*.

13. Run *Ovito_Figures.ipynb* and *Ovito_Figures_Post.ipynb*

***Note:*** While the code *Ovito_Figures.ipynb* renders the optimized supported nanoparticle configurations, the *Ovito_Figures_Post.ipynb* render figures colored depending on the Wigner-Seitz analysis.

14. Run *NP-Slab_Figures.ipynb,* followed by *Ovito_Eval.ipynb*

***Note:*** The notebook *NP-Slab_Figures.ipynb* generates two types of outputs. First, it creates charge distribution diagrams along the supported nanoparticle system, as shown in Figure 1C, and saves them to *Figures/Eval/ChargeDist.png*. Second, it produces the configurational map used to summarize the generated supported nanoparticle configurations, consistent with Figure 1D of the associated research article,^1^ and saves it to *All/Figures/Eval/SE_165-XX-Y-1.png*. The notebook *Ovito_Eval.ipynb* then combines the rendered structure images generated in the previous step using *Ovito_Figures.ipynb* with the charge distribution diagrams to create composite figures such as the one depicted in Figure 1C. To align the scatter points in the charge distribution diagram with the rendered structure view, adjust the following variables:

> dzCell = 26.75 # decreasing shifts lower points upward

> y_max = 41 # decreasing shifts upper points upward

Here, the plotted z coordinate is normalized by subtracting *dzCell*. Decreasing *dzCell* therefore shifts the entire point cloud upward, and increasing it shifts the cloud downward. The variable *y_max* defines the upper *z* limit of the plot. Decreasing *y_max* compresses the vertical axis range, moving the uppermost points of the cloud upward, and increasing it moves them downward. The values given above reproduce the reference case.

### Modeling and simulation for adsorption on supported nanoparticle

**Timing: 7 h**

**Figure 2:**
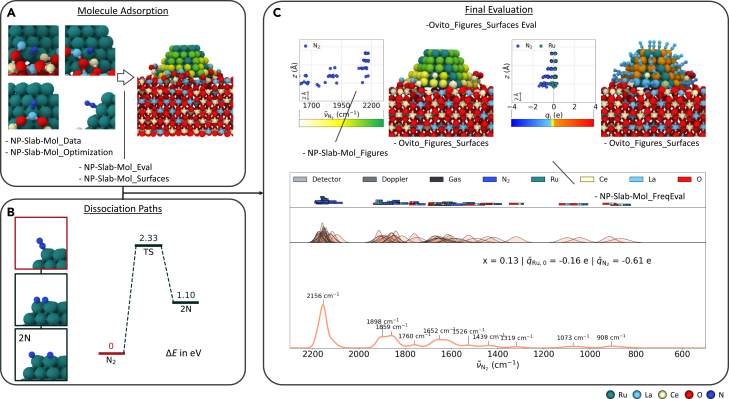
Workflow for adsorption, dissociation, and spectroscopic evaluation on supported nanoparticle catalysts (A) Generation and optimization of supported nanoparticle adsorption configurations. (B) Calculation of dissociation pathways and associated energy profiles for adsorbed molecules, illustrated by exemplary initial, transition, and final states. (C) Final evaluation and analysis, including analysis of spatially resolved surface property maps and vibrational frequency distribution.

Model and optimize the adsorption of homonuclear diatomic molecules on supported nanoparticles by relaxing all atomic positions to obtain stable adsorption configurations (see Figure 2A).

***Note:*** The working directory for this step is *SupplementaryDataP/InterF/SSF/NPSBMOL*/Diatom/. Moreover, the reported timing corresponds to the full modeling and geometry optimization workflow when evaluating all on top adsorption sites for a single catalyst model.

15. Run *NP-Slab-Mol_Data.ipynb*

***Note:*** The code is divided into two sections: one for preparing the molecule and one for assembling the molecule with the supported nanoparticle model into individual adsorption configurations. For molecule preparation, specifying the chemical element as

> element = 'N'

and changing it to the desired element is sufficient. The generation of adsorption configurations is controlled by the following variables:

 > case_ads = '”

 > dzAds = 1.6 # in Å

 > dxAds = 0.5 # in Å

 > dz = 1.4 # in Å

 > dz_factor = 1.0

 > kNew = range(0,10000)  # or a list such as [18,53,...]

Here, *case_ads* selects the adsorption geometry generated for each surface site, including several options (on-top: '', side-on right: ‘sr’, side-on left: ‘sl’, side-on at fourfold hollow sites: ‘4fsu′, and side-on at all bridge sites: ‘bs’). While the code supports the additional side-on adsorption configurations, they were not used in the associated research article.^1^ The variable *dzAds* defines the initial molecule–surface separation along the placement vector. For side-on placements (‘sr’ and ‘sl’), *dxAds* introduces a lateral offset. The variable *dz* shifts the reference point at which all placement vectors intersect, corresponding to the median nanoparticle position in the x and y directions and the minimum nanoparticle position along z, shifted by *dz* along the surface normal (see associated research article^1^ for details). The factor *dz_factor* controls the magnitude of *dz*. Increasing *dz_factor* typically results in a more perpendicular molecular orientation, whereas decreasing it yields flatter adsorption geometries. If unexpected behavior is observed during optimization, the corresponding adsorption configurations should be inspected and regenerated. This can be done efficiently by adjusting *globName* and setting *kNew* to a list of selected site indices (e.g., [18,53,…]), so that only the problematic configurations are rebuilt without regenerating the full dataset. A typical cause is an unfavorable initial molecular adsorption position (e.g., too close to a decoration atom), which can often be corrected by adjusting the parameters and rerunning the configuration generation for the affected configuration numbers. The models are saved as *NP-Slab-Mol.xyz*. In the reference case the path follows the form*/Ru-La-Ce-O-N/Ru238-La2Ce2O7-N2/…/X*, where *X* denotes the adsorption configuration number.

16. Run *NP-Slab-Mol_Optimization.ipynb*

***Note:*** In case of convergence issues with PFP, switching to the LBFGS optimizer may help:

> opt = LBFGS(NPSlabMoli, logfile=f'{rand_str}.log')

Otherwise, inspect the generated models for the problematic cases (see previous step’s Note). Optimized configurations are saved as *Opt_PFP.xyz*.

17. Run *NP-Slab-Mol_Eval.ipynb*

***Note:*** This step computes vibrational frequencies of the adsorbed molecule and evaluates a set of energetic, geometrical, and electronic properties. The behavior of the workflow is controlled by the variable:

> mode = 'eval'

The full analysis should be performed by setting mode to *‘eval’*, which combines energetic, vibrational, and geometrical evaluation in a single workflow and produces the complete set of output files. However, users may wish to modify parameters related to the geometrical evaluation without performing the computationally expensive calculations. In such cases, the analysis can be performed in a separated manner. Setting mode to *‘geom’* enables fast evaluation of geometrical descriptors only, while setting mode to *‘calc’* allows calculation of energetic and vibrational properties without repeating the geometrical analysis. For vibrational calculations, the normal modes of the adsorbed molecule are computed and saved as *Opt_Vib_PFP.xyz*. Separate JSON files are created for geometrical properties (*Opt_PFP_geom.json*) and calculated properties (*Opt_PFP_calc.json*). When the full evaluation is performed, all quantities are additionally saved as *Opt_PFP.json* and *Opt_PFP.csv*. The evaluation is further controlled by the following variables:

> MtName = “Ru”

> element = “N”

> massEl = None #massEl = 15.0001088989

> rcut = 10.5

> dxNPSlab_min = 2.3

> dxMol_min = 2.5

Here, *MtName* and *element* have the same meaning as in previous sections of the protocol. If *massEl* is set, an isotopic mass is assigned to the adsorbate atoms for the vibrational analysis. The cutoff *rcut* defines the maximum distance used to collect molecule–supported nanoparticle distance descriptors. The thresholds *dxNPSlab_min* and *dxMol_min* are applied during the final evaluation stage to automatically flag physically meaningful adsorption configurations, together with a minimum vibrational frequency criterion *freq_min*, defined as 1200 cm^-1^ for ^14^N and 1150 cm^-1^ for ^15^N, which can be adjusted as required (see associated research article^1^ for further details). Finally, *include_strVk* and *exclude_strVk* control which configuration subsets are evaluated. The list *include_strVk* must contain at least one substring present in the directory paths selected for evaluation.

18. Run *NP-Slab-Mol_Surfaces.ipynb*

***Note:*** This code generates XYZ files for spatial maps (refer to Figure 1E of the associated research article^1^). For each supported nanoparticle configuration, it loops over all adsorption sites and reads the per-site properties obtained in the previous step. The nanoparticle atom defining the adsorption site is relabeled using:

> elementRef = 'Ir'

to highlight them. The chosen element is arbitrary and should not be present in the system. Multiple XYZ files containing the mapped properties are written to the *Eval* directory. For example, adsorption energy maps are saved as *NP-Slab_eads.xyz* for each supported nanoparticle configuration. In addition, a thermodynamic analysis is performed. The temperature and pressure are provided as lists:

> TVk = [298.15,350+273.15] # in K

> pVk = [6000,1000000.1] # in Pa

Thermodynamic properties at these conditions are calculated and saved together with the XYZ files containing thermodynamic property spatial maps.

19. Run *Ovito_Figures.ipynb* and *Ovito_Figures_Surfaces.ipynb.*

***Note:*** The notebook *Ovito_Figures_Surfaces.ipynb* renders spatially resolved property maps on the supported nanoparticle surfaces. The list *fName* specifies the XYZ files containing the property maps and can be modified to control which spatial maps are rendered.

### Modeling and simulation of dissociation paths

**Timing: 1 h**

Model and simulate the dissociation of homonuclear diatomic molecules on supported nanoparticles with SMSI (see Figure 2B).

***Note:*** The reported timing corresponds to the full modeling and calculation workflow when evaluating a single on top adsorption site.

20. Prepare models for dissociated molecules on supported nanoparticles with SMSI and perform all-atom optimization.
  ***Note:*** The working directory for this step is *SupplementaryDataP/InterF/SSF/NPSBMOL/Atom/*
  a. Run *NP-Slab-2Atom_Data.ipynb*
    ***Note:*** This code follows the same logic as *NP-Slab-Mol_Data.ipynb* from the previous section. The variable *case_adsM* points to adsorption configurations generated in that step, while the variable
    > case_ads = 'RL' # 'RL', 'TB', 'BS' or 'B5'
    defines how the molecule is dissociated in the current workflow. Here, ‘TB’ corresponds to dissociation perpendicular to the surface, ‘RL’ to dissociation parallel to the surface, ‘BS’ to dissociation from a bridge site, and ‘B5’ to dissociation at a B5 site. The models are saved as *NP-Slab-Atom.xyz*, and their configuration numbering is kept consistent with the corresponding on-top adsorption site numbering of the molecular adsorption step, enabling direct pairing of adsorbed (initial) and dissociated states (end) in later reaction-path analyses.
  b. Run *NP-Slab-2Atom_Optimization.ipynb*
21. Perform reaction-path calculation
  ***Note:*** The working directory for this step is *SupplementaryDataP/InterF/SSF/NPSBMOL/Di-Atom*
  c. Run *NP-Slab-Mol-2Atom_Data.ipynb*
    ***Note:*** The code reads the molecular adsorption and dissociated configurations saved under the same adsorption configuration number and prepares three structures for reaction-path calculations: *Init.xyz* (adsorbed state), *End.xyz* (dissociated state), and *Mid.xyz* (an interpolated configuration). The interpolation can be adjusted using:
    > interp_factor = 0.5
  d. Run *NP-Slab-Mol-2Atom-DM.ipynb*
    ***Note:*** This code reads the configurations created in the last step and searches for transition states using the Dimer method.^25^ The following variables control the search:
    Here, *NIt* is the number of independent Dimer attempts started from the same *Mid.xyz* structure. *rCut* defines the cutoff radius in Å used to build the move mask around the adsorbate, so that atoms outside this distance are fixed. Furthermore, steps is the maximum number of optimization steps and *fMax* is the force convergence criterion for the Dimer search and the final FIRE^26^ optimization. Finally, *fMax_bfgsl* is the force threshold used for the initial forward relaxation with BFGS line search^6^ before switching to FIRE for final convergence toward the dissociated state. Various files are created during this step. *Init.xyz* and *End.xyz* are copied from the *Data* directory for reference. *TS_PFP.xyz* contains the transition-state structures that successfully converged. *End_Opt.xyz* stores the relaxation trajectories from the transition state toward the dissociated final state. The file *TS_Fix.csv* records which atoms were fixed during the Dimer and relaxation steps. All log files contain the corresponding optimizer and Dimer output.
    > NIt = 10
    > rCut = 3.0 # in Å
    > steps = 3000
    > fMax = 0.001 # in eV/Å
    > fMax_bfgsl = 0.005 # in eV/Å
  e. Run *NP-Slab-Mol-2Atom-NEB.ipynb*
    ***Note:*** This code performs reaction-path calculations using the Nudged Elastic Band (NEB) and climbing-image NEB (CI-NEB) methods. For each successful Dimer attempt, the reaction path is constructed separately using the corresponding initial adsorption configuration (*Init.xyz*), transition-state configuration (*TS_PFP.xyz*), and dissociated configuration (*End_Opt.xyz*) generated in the previous steps. The following variables control the calculation:
    Here, *NIt* defines the number of iterative NEB refinements starting from the same initial path. A value of 1 results in three images, a value of 2 in five images, and so on. The force convergence thresholds *fMaxNEB* and *fMaxCNEB* control the stopping criteria for the standard NEB relaxation and the climbing-image refinement, respectively. Atoms outside the reactive region are fixed using the mask defined in *TS_Fix.csv*, ensuring consistency with the preceding Dimer transition-state search. The resulting reaction-path trajectories are written as *NEBxN_XX.xyz* files in the Results directory, where N denotes the number of NEB refinement iterations and XX the image index along the reaction path. Detailed optimizer output for all NEB and CI-NEB runs is stored in NEB.log.
    > NIt = 2
    > fMaxNEB = 0.05 # in eV/Å
    > fMaxCNEB = 0.005 # in eV/Å
  f. Run *NP-Slab-Mol-2Atom-Eval.ipynb*, followed by *NP-Slab-Mol-2Atom-Thermo.ipynb*
    ***Note:*** These notebooks operate analogously to those used for molecular adsorption configurations. They perform vibrational analysis of the reaction-path configurations. Correspondingly, *NP-Slab-Mol-2Atom-Thermo.ipynb* evaluates finite-temperature and finite-pressure thermodynamic quantities based on the vibrational data. The results are written to CSV files.
  g. Run *NP-Slab-Mol-2Atom-Figures.ipynb* and *Ovito_Figures.ipynb.*

### Final evaluation and analysis

**Timing: 4 h**

Perform the final evaluation and analysis using the data generated in the previous sections (see Figure 2C).

***Note:*** The working directory for this step is *SupplementaryDataP/InterF/SSF/NPSBMOL/Diatom/*.

22. Prepare manually files containing experimental data from IR spectra.

***Note:*** The current implementation is based on JSON files that contain a vibrational frequency grid and the corresponding IR intensities for different experimental conditions. The JSON files used in the associated research article^1^ are provided as examples and are stored under *Doku/Eval/* as *IRSpectra_T25C-p6kPa.json*.

23. Run *NP-Slab-Mol_ReadData.ipynb*

***Note:*** This step reads all relevant evaluation and thermodynamic data generated in the previous sections and stores them in Python variables. All collected data are saved as serialized Python objects in the directory *Python/Vars* under file names starting with *NPSlabMolReadData*.

24. Run *NP-Slab-Mol_Figures.ipynb*

***Note:*** This notebook reads the dataset generated in the last step and creates figures based on the stored properties. Among others, example figures created in this step is Figure 2A.

25. Run *Ovito_Figures_Surfaces_Eval.ipynb*

***Note:*** This notebook combines figures generated in *NP-Slab-Mol_Figures.ipynb* with figures from the *Modeling & Simulation for Adsorption on Supported Nanoparticles* section (*Ovito_Figures_Surfaces.ipynb*) to create figures as depicted in Figures 2C and 2D of the associated research article.^1^

26. Run *NP-Slab-Mol_FreqEval.ipynb*

***Note:*** The dataset generated with *NP-Slab-Mol_ReadData.ipynb* is read and vibrational frequency evaluation is performed for all supported nanoparticle cases. For each catalyst configuration and thermodynamic condition, the notebook calculates the wavenumber distribution following the procedure described in Figure S30 of the associated research article^1^ and generates figures analogous to Figure 3A for all catalyst configurations. Users who wish to apply the method can find the relevant implementation enclosed between

> ###--- Wavenumber Distribution Calculation ---###

and

> ###--- End Wavenumber Distribution Calculation ---###

The generated variables are saved as serialized Python objects in *Python/Vars* under *NPSlabMolFreqEval.*

27. Run *NP-Slab-Mol_FreqFigs.ipynb*

***Note:*** In this final step, the matching of the wavenumber distributions with the experimental IR spectra is performed using a genetic algorithm, together with the similarity analysis described in detail in the Supplementary Information of the associated research article.^1^ The implementation of the genetic algorithm is located between

> ###--- GA Calculation ---###

and

> ###--- End GA Calculation ---###

The corresponding parameters are similarly marked between

> ###--- Parameters of the GA ---###

and

> ###--- End Parameters of the GA ---###

The functions implementing the genetic operators are read from *GA03_funcs.py*. In addition, this step includes the counting of highly active sites and the calculation of radial distribution functions (RDFs) (see Table 1 and Figure 4 of the associated research article^1^ for reference, respectively).

**CRITICAL:** While all preceding steps can be performed using the example data provided in the Supplementary Data, executing the final evaluation step with this reduced dataset will not yield results comparable to those reported in the associated research article.^1^ As a minimal working example, users may consider evaluating adsorption on a single support SS configuration across all considered reduction degrees and artificial SMSI models, including the different SMSI oxidation states. This may result in a dataset comprising 41 catalyst configurations and 3116 adsorption sites.

## Expected outcomes

By following this protocol, users obtain a practical and transferable workflow for modeling, optimizing, and analyzing supported nanoparticle catalysts exhibiting strong metal–support interactions (SMSI). Beyond the specific Ru/La_0_._5_Ce_0_._5_O_x-1_._75_ case study, the protocol can be directly applied to monometallic nanoparticles supported on similar oxide supports containing two cation types, oxygen vacancies, and reducible surfaces, where SMSI effects are expected to play a key role, provided that an accurate reactive force field with partial charges is available.

A central outcome of this protocol is a structured approach to modeling SMSI under realistic conditions. The workflow demonstrates how SMSI-induced nanoparticle decoration and support restructuring can be achieved through carefully designed optimization strategies, rather than relying on high-temperature molecular dynamics as used in another study.^27^ This approach facilitates the generation of large ensembles of catalyst configurations that are required to obtain statistically representative models consistent with experimental observations.

In addition, users gain a reusable framework for handling and visualizing large configurational datasets. The protocol demonstrates how to automatically generate publication-ready atomistic figures using Python and OVITO, including spatially resolved property maps, charge distributions, and combined structural and graphical representations. The workflow shows how such figures can be constructed reproducibly and consistently across large numbers of configurations, avoiding manual post-processing. This capability is particularly valuable for large datasets, where the systematic and error-free generation of comparable visual outputs would otherwise be time-consuming and impractical.

Finally, the protocol serves as a guide for implementing robust evaluation strategies, such as the matching of simulated vibrational signatures with experimental IR spectra. While demonstrated for a specific case study, these analysis concepts can be adapted to other systems to identify catalytically relevant configurations and active sites in complex supported nanoparticle catalysts.

## Limitations

This protocol is designed for use with reactive force fields that are compatible with the ASE, cover a broad range of elements, and provide access to partial charges. In the associated research article,^1^ these requirements are fulfilled by the PFP. For non-reactive force fields, dissociation processes cannot be described, and reaction pathways are therefore not accessible within this workflow. In addition, for other force fields it is generally unclear whether SMSI formation and support restructuring can be reliably reproduced. As a result, while individual steps of the protocol may still be applicable with alternative force fields, the full SMSI modeling workflow is not guaranteed to function as intended.

Furthermore, the workflow is provided as a research-oriented framework rather than a fully user-friendly software package. Several steps require direct code modification, parameter tuning, and system-specific decisions. Adapting the protocol to new material systems therefore requires solid Python programming skills and familiarity with ASE, and may benefit from consultation with the authors. Finally, while guidance on minimal dataset requirements for the final evaluation and genetic algorithm–based analysis is provided, the example dataset included here is primarily intended for demonstration purposes.

## Troubleshooting

### Problem 1

Depending on the ASE version, XYZ files may contain different numbers or types of columns. When rendering these files with OVITO (see steps 2c, 4c, 6, 13, 14, 19, 21 g und 25) this can lead to errors due to missing properties or mismatched property assignments (for example, Particle Type being linked to an unintended column).

### Potential solution

Check which properties and column order the XYZ files actually contain. Then adjust the *XYZProp* list in the code so that it matches the properties and their order in the XYZ file.

### Problem 2

During the reaction-path calculations (step 21), unphysical behavior may occur, such as unrealistic activation energies or the absence of a converged transition state. In some cases, this behavior appears systematically for all predefined dissociation end-state definitions (e.g., RL, TB).

### Potential solution

Create manually improved dissociated configurations based on chemically motivated structures from the literature or manual inspection. A practical approach is to store them in the directory corresponding to the desired adsorption-configuration number, so that they can be used consistently in subsequent reaction-path calculations.

## Resource availability

### Lead contact

Further information and requests for data and codes should be directed to and will be fulfilled by the lead contact, Gerardo Valadez Huerta (g.valadezhuerta@gmail.com).

### Technical contact

Technical questions on executing this protocol should be directed to and will be answered by the technical contact, Gerardo Valadez Huerta (g.valadezhuerta@gmail.com).

### Materials availability

No physical materials, chemicals, or biological reagents were used or generated in this study.

### Data and code availability

This protocol does not generate new code or large-scale datasets. The code used here is identical to that reported in the associated research article.^1^ However, it has been rearranged to be directly runnable and cleaned for clarity and reproducibility within this protocol. The curated version is available in a new Mendeley Data repository: https://doi.org/10.17632/ssk54nxjnr.1. The repository includes all components required to reproduce the workflow, including the custom Python module *vala_shinshu*. The version corresponding to this study is archived to ensure long-term accessibility and reproducibility. The repository includes newly generated minimal example data, provided as representative input and output files to enable users to run and test the workflow. However, these example data are intended for demonstration purposes and do not reproduce the full quantitative results of the associated research article.^1^ The complete datasets and full computational results produced using this protocol were originally reported in the associated research article.^1^ Any additional data or materials can be obtained from the lead contact upon reasonable request.

## Acknowledgments

This work was supported by the 10.13039/100005156Alexander von Humboldt Foundation (P20701) and the 10.13039/501100001691Japan Society for the Promotion of Science (JSPS) through 10.13039/501100001691KAKENHI grant nos. JP21F30701 and JP20H05623. Additional support was provided by the 10.13039/501100006120Ministry of the Environment, Japan, under the “Demonstration Project of Innovative Catalyst Technology for Decarbonization through Regional Resource Recycling.” No specific grant or funding number is assigned to this project.

## Author contributions

Conceptualization, G.V.H. and M.K.; methodology, G.V.H.; software, G.V.H.; validation, G.V.H.; formal analysis, G.V.H.; investigation, G.V.H.; resources, M.K.; data curation, G.V.H.; writing – original draft, G.V.H.; writing – review and editing, G.V.H. and M.K.; visualization, G.V.H.; supervision, M.K.; project administration, M.K.; funding acquisition, M.K.

## Declaration of interests

The authors declare no competing interests.
